# Flexible synthesis of poison-frog alkaloids of the 5,8-disubstituted indolizidine-class. II: Synthesis of (-)-**209B**, (-)-**231C**, (-)-**233D**, (-)-**235B"**, (-)-**221I**, and an epimer of **193E** and pharmacological effects at neuronal nicotinic acetylcholine receptors

**DOI:** 10.1186/1860-5397-3-30

**Published:** 2007-09-28

**Authors:** Soushi Kobayashi, Naoki Toyooka, Dejun Zhou, Hiroshi Tsuneki, Tsutomu Wada, Toshiyasu Sasaoka, Hideki Sakai, Hideo Nemoto, H Martin Garraffo, Thomas F Spande, John W Daly

**Affiliations:** 1 Graduate School of Medicine and Pharmaceutical Sciences, University of Toyama, Sugitani 2630, Toyama, 930-0194, Japan; 2 Laboratory of Bioorganic Chemistry, National Institute of Diabetes and Digestive and Kidney Diseases, National Institutes of Health, DHHS, Bethesda, MD 20892, USA

## Abstract

**Background:**

The 5,8-disubstituted indolizidines constitute the largest class of poison-frog alkaloids. Some alkaloids have been shown to act as noncompetitive blockers at nicotinic acetylcholine receptors but the proposed structures and the biological activities of most of the 5,8-disubstituted indolizidines have not been determined because of limited supplies of the natural products. We have therefore conducted experiments to confirm proposed structures and determine biological activities using synthetic compounds. Recently, we reported that one of this class of alkaloids, (-)-**235B'**, acts as a noncompetitive antagonist for α4β2 nicotinic receptors, and its sensitivity is comparable to that of the classical competitive antagonist for this receptor, dihydro-β-erythroidine.

**Results:**

The enantioselective syntheses of (-)-**209B**, (-)-**231C**, (-)-**233D**, (-)-**235B"**, (-)-**221I**, and what proved to be an epimer of natural **193E**, starting from common chiral lactams have been achieved. When we performed electrophysiological recordings to examine the effects of the synthetic alkaloids on two major subtypes of nicotinic receptors (α4β2 and α7) expressed in *Xenopus laevis* oocytes, (-)-**231C** effectively blocked α4β2 receptor responses (IC_50_ value, 1.5 μM) with a 7.0-fold higher potency than for blockade of α7 receptor responses. In contrast, synthetic (-)-**221I** and (-)-epi-**193E** were more potent in blocking α7 receptor responses (IC_50_ value, 4.4 μM and 9.1 μM, respectively) than α4β2 receptor responses (5.3-fold and 2.0-fold, respectively).

**Conclusion:**

We achieved the total synthesis of (-)-**209B**, (-)-**231C**, (-)-**233D**, (-)-**235B"**, (-)-**221I**, and an epimer of **193E** starting from common chiral lactams, and the absolute stereochemistry of natural (-)-**233D** was determined. Furthermore, the relative stereochemistry of (-)-**231C** and (-)-**221I** was also determined. The present asymmetric synthesis of the proposed structure for **193E** revealed that the C-8 configuration of natural **193E** should be revised. The selectivity for α4β2 and α7 nicotinic receptors differed markedly for the 5,8-disubstituted indolizidines tested, and thus it appears that the nature of the side chains in these indolizidines is crucial with regard to subtype-selectivity.

## Introduction

**Scheme 1:**
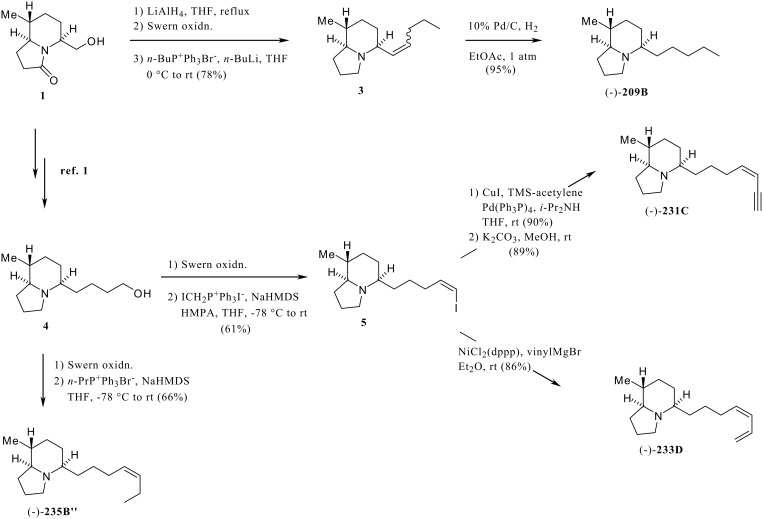
Syntheses of (-)-**209B**, (-)-**231C**, (-)-**233**D, and (-)-**235B"**.

**Scheme 2:**
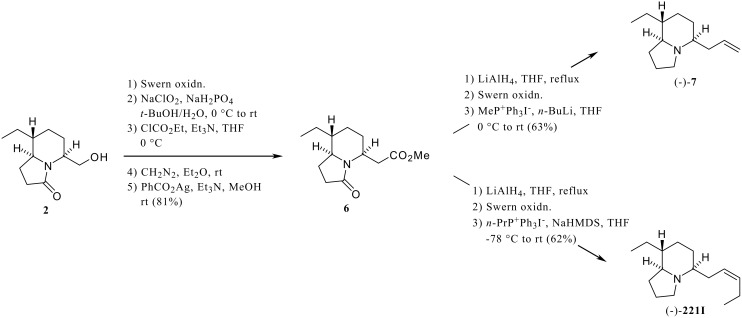
Syntheses of (-)-**221I** and (-)-**7** (an epimer of **193E**).

In the preceding paper [1], we have reported the synthesis of the chiral lactam building blocks (**1**, **2**, Scheme 1, Scheme 2) for the flexible synthesis of poison-frog alkaloids of the 5,8-disubstituted indolizidine class. The utility of these chiral building blocks was demonstrated by the synthesis of alkaloids (-)-**203A**, (-)-**205A** from **1**, and of (-)-**219F** from **2**. Although the biological activity of most of the 5,8-disubstituted indolizidines has not been investigated, certain 5,8-disubstituted indolizidines have been shown to act as noncompetitive blockers of nicotinic acetylcholine receptors. [2–3]

Nicotinic receptors are ligand-gated ion channels composed of five subunits. [4] To date, 12 nicotinic receptor subunits (α2-α10, β2-β4) have been identified. Subtypes of neuronal nicotinic receptors are constructed from numerous subunit combinations, which confer varied functional and pharmacological characteristics. [5] Nicotinic receptors have been implicated in a wide range of neuronal dysfunctions and mental illness, such as epilepsy, Tourette's syndrome, Alzheimer's disease, Parkinson's disease, and schizophrenia. [5–6] Since different subtypes of nicotinic receptors are involved in different neurological disorders, subtype-selective nicotinic ligands would be valuable for investigation and potentially for treatment of cholinergic disorders of the central nervous system. However, there are only a limited number of compounds that elicit subtype-selective blockade of nicotinic receptors because of the similarity of receptor-channel structure among the subtypes. Recently, we have investigated the effect of synthetic (-)-**235B'**, one of the 5,8-disubstituted indolizidine class of poison-frog alkaloids, on several subtypes of nicotinic receptors, and found that this alkaloid exhibits selective and potent blocking effects at the α4β2 nicotinic receptor. [3] The potency of (-)-**235B'** for this receptor is comparable to that of the classical α4β2 competitive antagonist, dihydro-β-erythroidine. In this study, we have synthesized 5,8-disubstituted indolizidines (-)-**209B**, (-)-**231C**, (-)-**233D**, (-)-**235B"**, (-)-**221I**, and an alkaloid that proved to be an epimer of natural indolizidine **193E**. The alkaloids (-)-**209B** and (-)-**235B"** are known to be noncompetitive nicotinic blockers [2], but effects of the other compounds have not yet been tested. To explore possible subtype selectivity, we examined the effects of (-)-**231C**, (-)-**221I** and (-)-epi-**193E** on α4β2 and α7 nicotinic receptors, the most abundant subtypes in the mammalian brain. [4]

## Results and Discussion

Reduction of the lactam **1** [1] with LiAlH_4_ followed by Swern oxidation of the resulting alcohol and Wittig reaction gave the olefin **3** in 78% overall yield (Scheme 1). Hydrogenation of the double bond in **3** with 10% Pd/C provided (-)-**209B**, whose spectral data were identical with reported values. [7] The lactam **1** was also converted to the alcohol **4**, [1] which was transformed into (-)-**235B"** by Swern oxidation followed by Wittig reaction under high dilution and 'salt free' conditions (Scheme 1). The spectral data of synthetic (-)-**235B"** were identical with reported values. [8–9] Indolizidines (-)-**231C** [10] and (-)-**233D** [10] were synthesized from common intermediate **5** prepared from the alcohol **4**. Thus, the Swern oxidation of **4** and then the Wittig reaction of the resulting aldehyde under Stork's conditions [11] provided the *Z*-iodoolefin **5** in a highly stereoselective manner. The Sonogashira coupling reaction [12] of **5** with TMS-acetylene followed by cleavage of the trimethylsilyl group with K_2_CO_3_ afforded (-)-**231C**. Although the rotation of the natural alkaloid is unknown, the relative stereochemistry was determined to be 5,8-*E* and 5,9-*Z* by GC-MS and GC-FTIR comparison with natural **231C** in extracts from a Panamanian dendrobatid frog, *Dendrolbates pumilio*. A similar, Ni-catalyzed cross coupling [13] reaction of **5** with vinylmagnesium bromide provided the (-)-**233D**, whose spectral data were identical with values reported for the natural alkaloid isolated from the Panamanian dendrobatid frog. [10] Although differing in magnitude, the HCl salts of both synthetic (-) **233D** and the natural alkaloid had negative optical rotations.

Indolizidine (-)-**7** with the relative stereochemistry proposed for **193E** [14] and indolizidine (-)-**221I** [14] were synthesized from the lactam **2** [1] via the ester **6** (Scheme 2). The two-step oxidation of **2** followed by Arndt-Eistert homologation of the resulting carboxylic acid provided the ester **6**. Reduction of both lactam and ester moieties of **6** with LiAlH_4_ followed by Swern oxidation and Wittig reaction of the resulting aldehyde furnished the indolizidine (-)-**7**. Coinjections of synthetic material with an alkaloid fraction from a Madagascan mantellid frog, *Mantella viridis* that contained natural **193E**, revealed that the synthetic material had a slightly longer GC retention time than the natural product. The GC-mass spectra of (-)-**7** and natural product were virtually identical and their GC-FTIR spectra were very similar in the Bohlmann band region (indicating 5,9-*Z* configurations in both), although differing slightly in their fingerprint regions. These results indicate that the natural **193E** is most likely the 8-epimer of (-)-**7** and that the proposed configuration [14] of the ethyl substituent at C-8 was in error. The indolizidine (-)-**221I** was also synthesized from **6** following a procedure similar to that used for the synthesis of (-)-**7** as shown in Scheme 2.

The relative stereochemistry of natural **221I** was determined to be the same as that of synthetic (-)-**221I** by GC-MS and GC-FTIR comparison with natural **221I**, in the alkaloid fraction from the Madagascan mantellid frog, *Mantella viridis* (See Supporting Information File 1 for experimental details relating to all syntheses).

**Figure 1:**
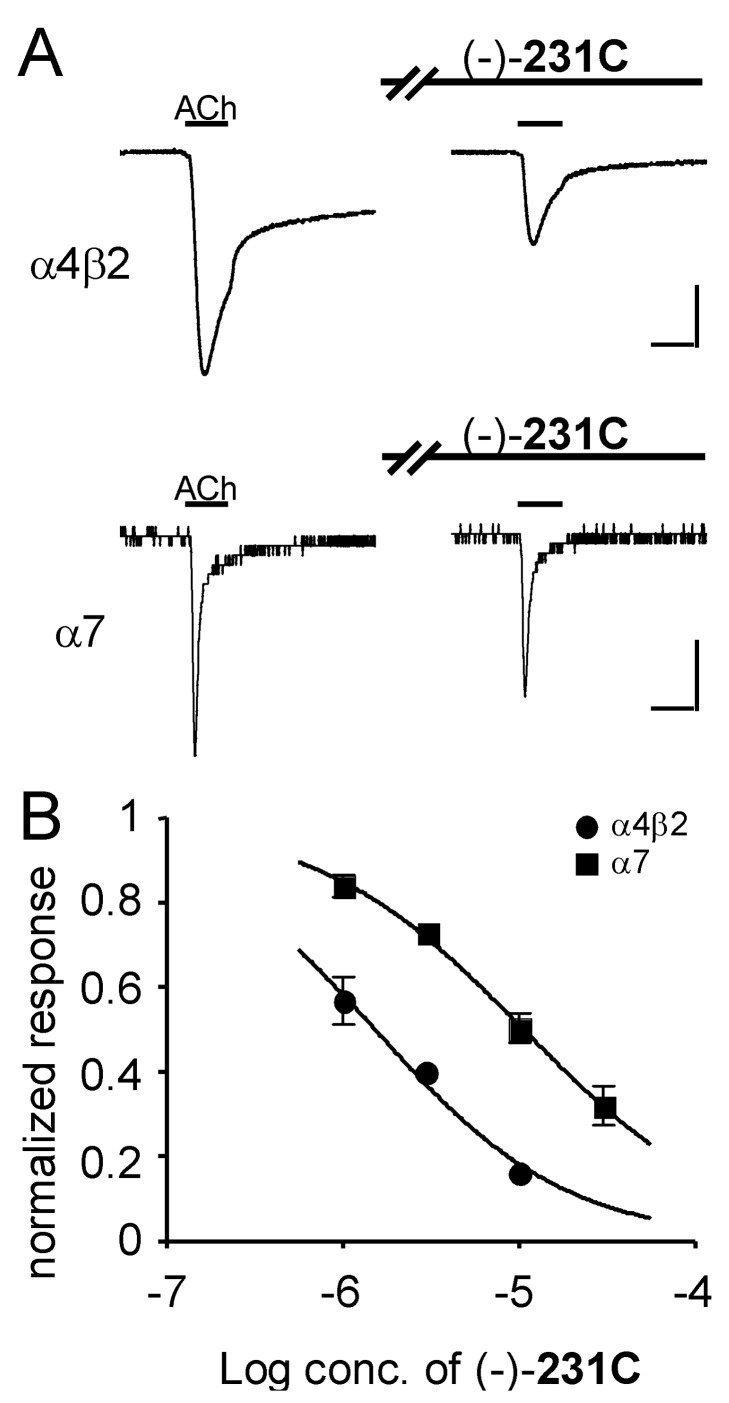
Inhibitory effect of (-)-**231C** on ACh-induced currents in *X. laevis* oocytes expressing recombinant nicotinic receptors. Currents were recorded in the voltage-clamp mode at -60 mV. Concentrations of ACh used were 1 μM for the α4β2 receptor and 100 μM for the α7 receptor. For test responses, oocytes were preincubated with (-)-**231C** for 3 min and then exposed to ACh with (-)-**231C**. A, representative traces showing the ACh-elicited currents in the absence and presence of (-)-**231C** (3 μM). Horizontal bars indicate the period of perfusion with ACh for 5 s. Vertical scale bars represent 0.5 μA on the α4β2 receptor, and 0.1 μA on the α7 receptor. B, concentration-response curves for (-)-**231C** on recombinant nicotinic receptors. Current responses to ACh in the presence of (-)-**231C** in each oocyte were normalized to the ACh responses (control responses) recorded in the same oocytes. Values represent the mean ± S.E.M. for five to six separate experiments.

We then conducted electrophysiological experiments to examine the effect of three of the synthetic alkaloids on nicotinic receptors. When *Xenopus laevis* oocytes expressing the α4β2 nicotinic receptor were treated with 3 μM (-)-**231C**, the peak amplitude of the acetylcholine (ACh)-elicited currents was greatly decreased, whereas the responses elicited in oocytes expressing the α7 nicotinic receptor were not strongly affected (Figure 1A). When the concentration-response curves were compared between these receptor subtypes, (-)-**231C** blocked the α4β2 receptor-mediated currents [50% inhibitory concentration (IC_50_) = 1.5 μM, 95% confidence intervals (CI): 1.1 to 2.1 μM] with 7.0-fold higher sensitivity than blockade of the α7 receptor-mediated currents (IC_50_ = 10.7 μM, 95% CI: 8.6 to 13.3 μM) (Figure 1B). These results indicate that (-)-**231C** selectively blocked the responses mediated by the α4β2 receptor.

The 5,8-disubstituted indolizidine (-)-**231C** is an analogue of (-)-**235B'**, both of which have a seven-carbon unsaturated side-chain at C-5 and a methyl at C-8. Both synthetic compounds have the same absolute stereochemistry (5*R*, 8*R*, 9*S*). Our previous [3] and present data demonstrate that both (-)-**235B'** and (-)-**231C** produce potent blockade of the α4β2 nicotinic receptor with a similar selectivity of 6- to 7-fold over blockade of the α7 receptor. However, the potency of (-)-**235B'** in blocking the α4β2 receptor is approximately 20-fold greater than that of (-)-**231C**. These results suggest that either flexibility or degree of unsaturation of the seven-carbon side-chain at C-5 in these 5,8-disubstituted indolizidines is crucial for potent interaction with the α4β2 receptor.

**Figure 2:**
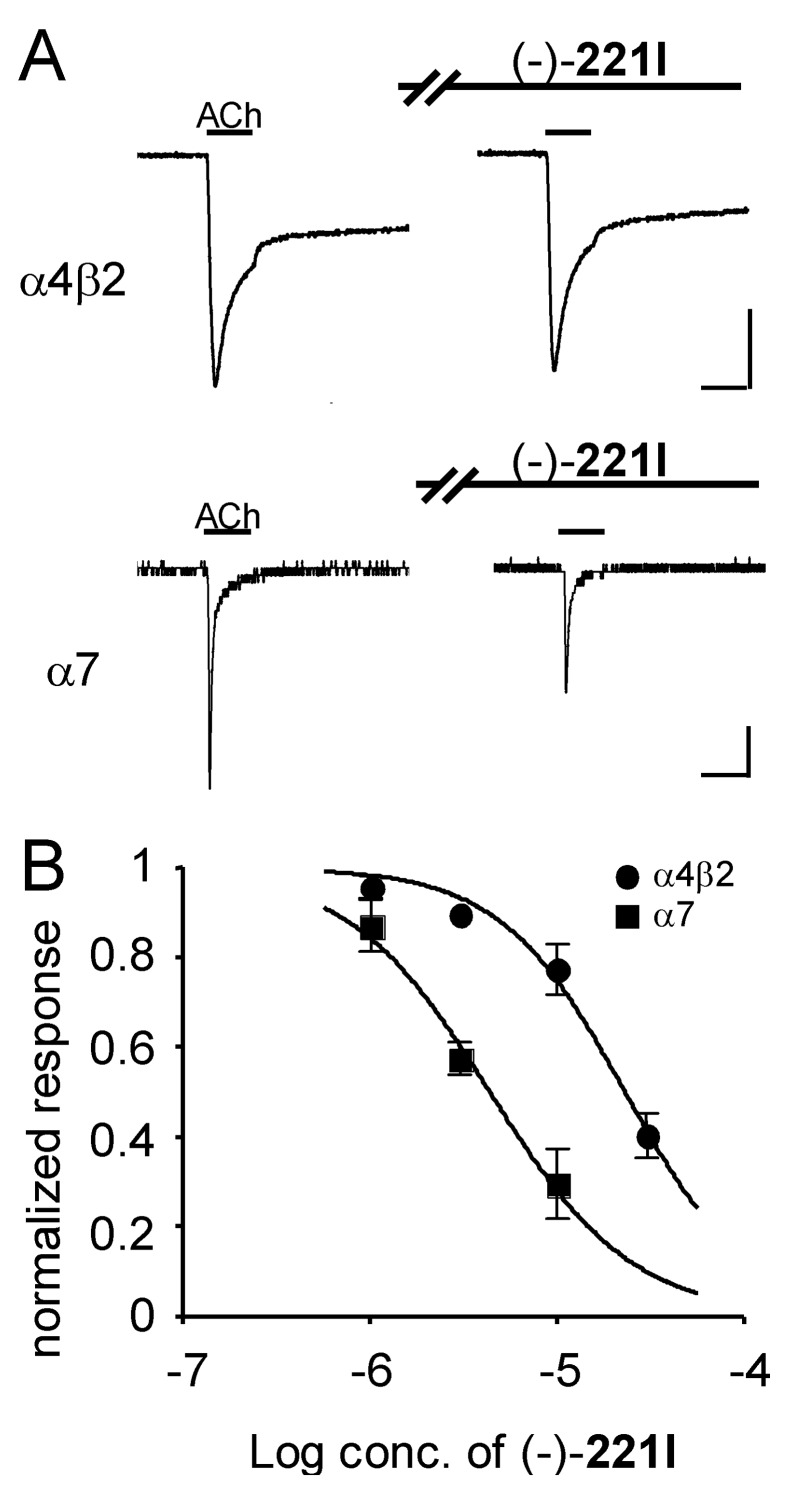
Inhibitory effect of (-)-**221I** on ACh-induced currents in *X. laevis* oocytes expressing recombinant nicotinic receptors. Currents were recorded in the voltage-clamp mode at -60 mV. Concentrations of ACh used were 1 μM for the α4β2 receptor and 100 μM for the α7 receptor. For test responses, oocytes were preincubated with (-)-**221I** for 3 min and then exposed to ACh with (-)-**221I**. A, representative traces showing the ACh-elicited currents in the absence and presence of (-)-**221I** (3 μM). Horizontal bars indicate the period of perfusion with ACh for 5 s. Vertical scale bars represent 0.5 μA on the α4β2 receptor, and 0.1 μA on the α7 receptor. B, concentration-response curves for (-)-**221I** on recombinant nicotinic receptors. Current responses to ACh in the presence of (-)-**221I** in each oocyte were normalized to the ACh responses (control responses) recorded in the same oocytes. Values represent the mean ± S.E.M. for five separate experiments.

**Figure 3:**
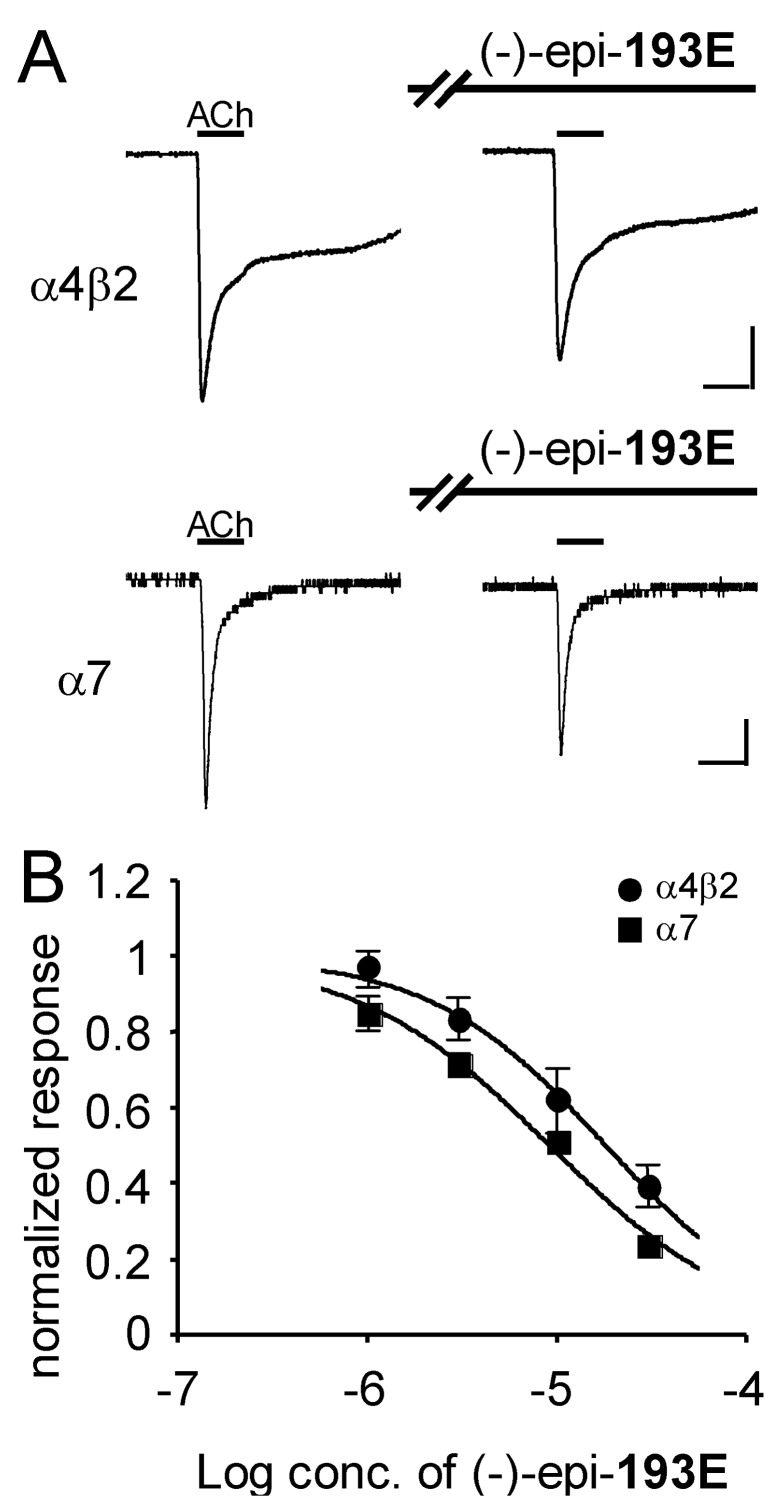
Inhibitory effect of (-)-epi-**193E** on ACh-induced currents in *X. laevis* oocytes expressing recombinant nicotinic receptors. Currents were recorded in the voltage-clamp mode at -60 mV. Concentrations of ACh used were 1 μM for the α4β2 receptor and 100 μM for the α7 receptor. For test responses, oocytes were preincubated with (-)-epi-**193E** for 3 min and then exposed to ACh with (-)-epi-**193E**. A, representative traces showing the ACh-elicited currents in the absence and presence of (-)-epi-**193E** (3 μM). Horizontal bars indicate the period of perfusion with ACh for 5 s. Vertical scale bars represent 0.5 μA on the α4β2 receptor, and 0.1 μA on the α7 receptor. B, concentration-response curves for (-)-epi-**193E** on recombinant nicotinic receptors. Current responses to ACh in the presence of (-)-epi-**193E** in each oocyte were normalized to the ACh responses (control responses) recorded in the same oocytes. Values represent the mean ± S.E.M. for five separate experiments.

The synthetic (-)-**221I** and (-)-epi-**193E** are 5,8-disubstituted indolizidines with an ethyl rather than a methyl at C-8 and a five-carbon or three-carbon side-chain, respectively, at C-5. The alkaloid (-)-**221I** blocked α7 receptor responses (IC_50_ = 4.4 μM, 95% CI: 3.1 to 6.1 μM) with 5.3-fold higher potency than for blockade of the α4β2 receptor responses (IC_50_ = 23.1 μM, 95% CI: 18.5 to 28.9 μM) (Figure 2). Synthetic (-)-epi-**193E** was more potent in blocking the α7 receptor response (IC_50_ = 9.1 μM, 95% CI: 7.5 to 11.1 μM) compared to blockade of the α4β2 receptor (IC_50_ = 18.0 μM, 95% CI: 12.2 to 26.7 μM) (Figure 3). Previously, we examined the effects of three synthetic 5,8-disubstituted indolizidines with an *n*-butyl group at C-8 and an *n*-propyl group at C-5 in blocking different subtypes of nicotinic receptors. [3] Two of these compounds, namely (+)-8,9-diepi-**223V** and (-)-9-epi-**223V** were 6.7-fold and 11.2-fold more potent in blocking α7 receptor compared to blockade of α4β2 receptor, while the third, (-) **223V**, was only slightly more potent at blocking the responses mediated by the α7 receptor. [3,15] These results suggest that the α4β2 receptor does not interact well with indolizidines having substituents larger than methyl at C-8, while the α7 receptor is more accepting of larger side-chains at C-8. Further analogous synthetic alkaloids need to be tested. Overall, the side chains of 5,8-disubstituted indolizidines appear to be of critical importance in determining selectivity and potency in blocking responses mediated by subtypes of neuronal nicotinic receptors. Further study of structure-activity relationships of synthetic 5,8-disubstituted indolizidines at nicotinic subtypes could lead to even more subtype-selective ligands as research probes and as potentially useful drugs.

Neuronal nicotinic receptors have been implicated in the physiological processes of reward, cognition, learning and memory. [5–6] Some ligand-binding and autoradiography studies with postmortem human brain suggest that loss of neuronal nicotinic receptors is related to central cholinergic disorders such as Alzheimer's disease, Parkinson's disease and schizophrenia. [4,6] For instance, in schizophrenic patients, decrease in binding of α-bungarotoxin (α-Bgt), a major specific ligand for α7 nicotinic receptors, has been detected in hippocampus, thalamus and frontal cortex [16–17]. Therefore, loss of α7 nicotinic ligand-binding appears to be an early presymptomatic diagnostic marker for schizophrenia. For *in vivo* mapping of brain receptors, positron emission tomography (PET) and single photon emission computed tomography (SPECT) using specific ligands are powerful, non-invasive techniques. Although ^125^I-methyllycaconitine has been used for α7-selective binding in rat brain, [18] neither PET nor SPECT ligand of α7 nicotinic receptors has been available so far. Radiolabeled α-Bgt could not be used for *in vivo* mapping because of the large molecular weight, high toxicity and poor blood-brain barrier permeability. [19] Indolizidines are low molecular weight, lipophilic compounds that should penetrate well into brain and, as shown in our research, some exhibit high affinity and selectivity for either α4β2 or α7 nicotinic receptors. Further structure-activity relationship studies of synthetic indolizidines may lead to the development of radioactive α4β2-selective or α7-selective ligands useful for *in vivo* mapping of these important central nicotinic receptors.

## Supporting Information

File 1 Experimental details for the synthesis of (-)-**209B**, (-)-**231C**, (-)-**233D**, (-)-**235B"**, (-)-**221I**, and an epimer of **193E** and pharmacological effects at neuronal nicotinic acetylcholine receptors. Experimental data which includes experimental details on the spectral instruments, elemental analyzer.
