# Supplementary material for: Flexible synthesis of poison-frog alkaloids of the 5,8-disubstituted indolizidine-class. II: Synthesis of (-)-209B, (-)-231C, (-)-233D, (-)-235B", (-)-221I, and an epimer of 193E and pharmacological effects at neuronal nicotinic acetylcholine receptors
Source: Beilstein J Org Chem. 2007 Sep 28;3:30. doi: 10.1186/1860-5397-3-30 (PMC2164953; doi:10.1186/1860-5397-3-30)
Supplement: File 1 — Experimental details for the synthesis of (-)-209B, (-)-231C, (-)-233D, (-)-235B", (-)-221I, and an epimer of 193E and pharmacological effects at neuronal nicotinic acetylcholine receptors. Experimental data which includes experimental details on the spectral instruments, elemental analyzer. [file Beilstein_J_Org_Chem-03-30-s001.doc]

**Experimental Details**

**General**

Melting points were determined with a Yanaco micro melting point apparatus and are uncorrected. ^1^H and ^13^C NMR spectra were taken on a Varian Gemini 300 or Unity Plus 500 spectrometer. ^1^H NMR spectra were recorded at the indicated field strength as solutions in CDCl_3_ unless otherwise indicated. Chemical shifts are given in parts per million (ppm, δ) downfield from TMS and are referenced to CHCl_3_ (7.26 ppm) as an internal standard. Splitting patterns are designated as s, singlet; d, doublet; t, triplet; q, quartet; m, multiplet; br, broad. ^13^C NMR spectra were recorded at the indicated field strength as solutions in CDCl_3_ unless otherwise indicated. Chemical shifts are given in ppm, (δ) downfield from TMS and are referenced to the center line of CDCl_3_ (77.0 ppm) as an internal standard. Carbon signals were assigned by a DEPT pulse sequence, q = methyl, t = methylene, d = methane, and s = quaternary carbons. Infrared spectra (IR) were measured with a Perkin-Elmer 1600 series FT-IR spectrophotometer. Mass spectra (MS) and high-resolution mass spectra (HRMS) were measured on a JEOL JMS-AX505HAD mass spectrometer. Optical rotations were measured on a JASCO DIP-1000 digital polarimeter. Column chromatography was performed with Kanto Kagaku silica gel 60N. GC-MS was performed with a Finnigan GCQ instrument fitted with a Restek Amine5x column (30 cmX0.25 mm) and a program 100 °C (1 min hold time) to 280 °C at 10 °C. GC-FTIR spectra were obtained with a narrow band HP model 5981 GC-FTIR infrared spectrophotometer interfaced with both an HP MSD, model 5971 and an HP 5890 gas chromatograph using the same temperature program as above and fitted with the same column except 0.32 mm i. d.

**(5*S*,8*R*,9*S*)-8-Methyl-5-pent-1-enyloctahydroindolizine (3)**

To a stirred solution of **1** (65 mg, 0.36 mmol) in THF (10 mL) was added LiAlH_4_ (40 mg, 1.07 mmol) at 0 °C, and the resulting suspension was refluxed for 13 h. After cooling, the reaction was quenched with 10% NaOH (aq), and the aqueous mixture was extracted with hot CHCl_3_ (10 mL x 10). The organic extracts were combined, dried over K_2_CO_3_, and evaporated to give a colorless oil, which was used directly in the next step.

To a stirred solution of (COCl)_2_ (0.1 mL, 1.14 mmol) in CH_2_Cl_2_ (1 mL) was added DMSO (0.17 mL, 2.40 mmol) at –78 °C, and the reaction mixture was stirred at –78 °C for 5 min. To the resulting mixture was added dropwise a solution of the alcohol obtained above in CH_2_Cl_2_ (2 mL) via a double-tipped stainless steel needle at –78 °C, and the reaction mixture was stirred at –78 °C for 30 min. Triethylamine (0.5 mL, 3.61 mmol) was added to the reaction mixture, and the reaction mixture was warmed to 0 °C for 1 h. The reaction was quenched with satd. NaHCO_3_ (aq), and the aqueous mixture was extracted with CH_2_Cl_2_ (10 mL x 3). The organic layers were combined, dried over K_2_CO_3_, and evaporated to give a pale yellow oil, which was used directly in the next step.

To a stirred suspension of *n*-BuP^+^Ph_3_Br^-^ (708 mg, 1.78 mmol) in THF (5 mL) was added a solution of *n*-BuLi (1 M in hexane, 1.00 mL, 1.60 mmol) at 0 °C, and the resulting mixture was stirred at the same temperature for 5 min. To the reaction mixture was added dropwise a solution of the aldehyde obtained above in THF (3 mL) via a double-tipped stainless steel needle at 0 °C. The reaction mixture was stirred at 0 °C~room temperature for 16 h., and the reaction was quenched with satd. NaHCO_3_ (aq). The aqueous mixture was extracted with Et_2_O (15 mL x 4), and the organic exracts were combined, dried over K_2_CO_3_, and evaporated to give a pale yellow oil, which was chromatographed on silica gel (10 g, hexane : acetone = 100 : 1~20 : 1) to afford **3** (57 mg, 78%) as a pale yellow oil.

^1^H NMR (500 MHz) δ 0.88 (3H, d, *J* = 6.0 Hz), 0.92 (3H, t, *J* = 7.7 Hz), 0.99-1.08 (2H, m), 1.20-1.52 (6H, br m), 1.54-1.62 (2H, m), 1.68-1.77 (2H, m), 1.94-2.05 (2H, m), 2.06-2.13 (2H, m), 2.82 (1H, br), 3.17 (1H, t-like, *J* = 6.6 Hz), 5.40-5.44 (2H, m).

**(5*S*,8*R*,9*S*)-(-)-8-Methyl-5-pentyloctahydroindolizine (209B)**

To a stirred solution of **3** (57 mg, 0.28 mmol) in EtOAc (10 mL) was added 10% Pd/C (20 mg), and the resulting suspension was hydrogenated under hydrogen atmosphere at 1 atm for 20 h. The catalyst was removed by filtration and the filtrate was evaporated to give **209B** (55 mg, 95%) as a colorless oil.

IR (neat) 2955, 2929, 2862, 2775, 2701, 1457 cm^-1^; ^1^H NMR (500 MHz) δ 0.85 (3H, d, *J* = 6.4 Hz), 0.88 (3H, t, *J* = 7.0 Hz), 0.95 (1H, m), 1.18-1.52 (11H, br m), 1.60-1.68 (2H, m), 1.71-1.78 (3H, m), 1.83-1.97 (2H, m), 1.98 (1H, q-like, *J* = 8.9 Hz), 3.27 (1H, t-like, *J* = 8.7 Hz); ^13^C NMR (75 MHz) δ 14.18 (q), 18.98 (q), 20.40 (t), 22.72 (t), 25.61 (t), 29.09 (t), 31.24 (t), 32.34 (t), 33.74 (t), 34.59 (t), 36.55 (d), 51.84 (t), 63.59 (d), 71.34 (d); MS: 209 (M^+^); [α]_D_^26^ –91.7 (*c* 1.06, MeOH)., Lit. [α]_D_^28^ –91.3 (*c* 0.58, MeOH).

**(5*R*,8*R*,9*S*)-(-)-5-Hept-4-enyl-8-methyloctahydroindolizine (235B’’)**

To a stirred solution of (COCl)_2_ (0.1 mL, 1.14 mmol) in CH_2_Cl_2_ (1 mL) was added DMSO (0.17 mL, 2.40 mmol) at –78 °C, and the reaction mixture was stirred at –78 °C for 5 min. To the resulting mixture was added dropwise a solution of **4** (60 mg, 0.28 mmol) in CH_2_Cl_2_ (2 mL) via a double-tipped stainless steel needle at –78 °C, and the reaction mixture was stirred at –78 °C for 30 min. Triethylamine (0.5 mL, 3.61 mmol) was added to the reaction mixture, and the reaction mixture was warmed to 0 °C for 1 h. The reaction was quenched with satd. NaHCO_3_ (aq) and the aqueous mixture was extracted with CH_2_Cl_2_ (10 mL x 3). The organic layers were combined, dried over K_2_CO_3_, and evaporated to give a pale yellow oil, which was used directly in the next step.

To a stirred suspension of *n*-PrP^+^Ph_3_Br^-^ (383 mg, 1.00 mmol) in THF (20 mL) was added a solution of NaHMDS (1 M in THF, 0.71 mL, 0.71 mmol) at room temperature, and the resulting mixture was stirred at the same temperature for 1 min. To the reaction mixture were added HMPA (0.5 mL), and then dropwise a solution of the aldehyde obtained above in THF (3 mL) via a double-tipped stainless steel needle at –78 °C. The reaction mixture was stirred at –78 °C~room temperature for 2 h. and the reaction was quenched with satd. NaHCO_3_ (aq). The aqueous mixture was extracted with Et_2_O (15 mL x 4), and the organic exracts were combined, dried over K_2_CO_3_, and evaporated to give a pale yellow oil, which was chromatographed on silica gel (10 g, hexane : acetone = 100 : 1~20 : 1) to afford **235B’’** (44 mg, 66%) as a colorless oil.

IR (neat) 3003, 2963, 2935, 2874, 2775, 2704, 1455 cm^-1^; ^1^H NMR (500 MHz) δ 0.85 (3H, d, *J* = 6.4 Hz), 0.90-0.98 (1H, m), 0.96 (3H, t, *J* = 7.7 Hz), 1.20-1.37 (5H, m), 1.41-1.55 (3H, m), 1.61-1.78 (5H, m), 1.86-2.07 (6H, br m), 3.28 (1H, t-like, *J* = 7.3 Hz), 5.29-5.39 (2H, m); ^13^C NMR (75 MHz) δ 14.47 (q), 18.96 (q), 20.36 (t), 20.61 (t), 26.07 (t), 27.41 (t), 29.00 (t), 31.10 (t), 33.66 (t), 34.11 (t), 36.36 (d), 51.83 (t), 63.56 (d), 71.42 (d), 128.76 (d), 131.70 (d); MS: 235 (M^+^); [α]_D_^26^ –80.9 (*c* 1.71, MeOH)., Lit. [α]_D_^28^ –85.4 (*c* 0.79, MeOH), Lit. [α]_D_^24^ –88.0 (*c* 1.0, MeOH).

**(5*R*,8*R*,9*S*)-(-)-5-(5-Iodopent-4-enyl)-8-methyloctahydroindolizine (5)**

To a stirred solution of (COCl)_2_ (0.35 mL, 4 mmol) in CH_2_Cl_2_ (10 mL) was added DMSO (0.58 mL, 8 mmol) at –78 °C, and the resulting mixture was stirred at the same temperature for 5 min. To the reaction mixture was added dropwise a solution of **4** (210 mg, 1.00 mmol) in CH_2_Cl_2_ (3 mL) via a double-tipped stainless steel needle at –78 °C, and the reaction mixture was stirred at –78 °C for 30 min. Triethylamine (1.66 mL, 12 mmol) was added to the reaction mixture, and the reaction mixture was warmed to 0 °C for 1 h. The reaction was quenched with satd. NaHCO_3_ (aq), and the aqueous mixture was extracted with CH_2_Cl_2_ (20 mL x 3). The organic layers were combined, dried over K_2_CO_3_, and evaporated to give a pale yellow oil, which was used directly in the next step.

To a stirred suspension of ICH_2_P^+^Ph_3_I^-^ (1.33 g, 2.5 mmol) in THF (15 mL) was added a solution of NaHMDS (1M in THF, 2.5 mL, 2.5 mmol) at room temperature, and the reaction mixture was stirred at room temperature for 1 min. To the reaction mixture were added HMPA (0.6 mL) and then dropwise a solution of the above aldehyde in THF (6 mL) via a double-tipped stainless steel needle at –78 °C. The reaction mixture was stirred at –78 °C~room temperature for 2 h. and quenched with satd. NaHCO_3_ (aq). The aqueous mixture was extracted with Et_2_O (25 mL x 4), and the organic extracts were combined, dried over K_2_CO_3_, and evaporated to give a pale yellow oil, which was chromatographed on silica gel (30 g, hexane : acetone = 80 : 1~20 : 1) to afford **5** (203 mg, 61%) as a pale yellow oil.

IR (neat) 2926, 2869, 2778, 2457 cm^-1^; ^1^H NMR (500 MHz) δ 0.88 (3H, d, *J* = 6.8 Hz), 0.96 (1H, qd-like, *J* = 12.0, 4.2 Hz), 1.22-1.41 (5H, br m), 1.42-1.58 (2H, m), 1.61-1.78 (5H, m), 1.89-1.95 (2H, m), 1.97 (1H, q-like, *J* = 8.9 Hz), 2.12-2.17 (2H, m), 3.26 (1H, t-like, *J* = 8.6 Hz), 6.15-6.21 (2H, m); ^13^C NMR (75 MHz) δ 18.96 (q), 20.42 (t), 24.22 (t), 29.09 (t), 31.18 (t), 33.67 (t), 34.04 (t), 35.00 (t), 36.57 (d), 51.86 (t), 63.17 (d), 71.31 (d), 82.50 (d), 140.96 (d); MS: 333 (M^+^); HRMS: Calcd for C_14_H_24_IN 333.0953; Found 333.0973; [α]_D_^26^ –67.4 (*c* 1.67, CHCl_3_).

**(5*R*,8*R*,9*S*)-(-)-8-Methyl-5-[7-(trimethylsilyl)hept-4-en-6-ynyl]octahydroindolizine**

To a stirred suspension of CuI (11 mg, 0.057 mmol) and Pd(Ph_3_P)_4_ (23 mg, 0.02 mmol) in THF (2 mL) was added *i*-Pr_2_NH (0.2 mL, 1.44 mmol) at room temperature. To the resulting solution was added dropwise a solution of **5** (65 mg, 0.2 mmol) and TMS-acetylene (0.1 mL, 0.93 mmol) in THF (3 mL) via a double-tipped stainless steel needle. The reaction mixture was stirred at room temperature for 17 h., and then quenched with satd. NaHCO_3_ (aq). The aqueous mixture was extracted with Et_2_O (10 mL x 4), and the organic extracts were combined, dried over K_2_CO_3_, and evaporated to give a pale yellow oil, which was chromatographed on silica gel (10 g, hexane : acetone = 100 : 1~20 : 1) to afford the acetylene (41 mg, 90%) as a pale yellow oil.

IR (neat) 3017, 2958, 2778, 2148, 843 cm^-1^; ^1^H NMR (500 MHz) δ 0.21 (9H, s), 0.88 (3H, d, *J* = 6.4 Hz), 0.97 (1H, qd-like, *J* = 12.0, 3.9 Hz), 1.27-1.53 (8H, br m), 1.67-1.78 (4H, m), 1.94-2.18 (3H, m), 2.33 (2H, q-like, *J* = 6.9 Hz), 3.29 (1H, br), 5.49 (1H, d-like, *J* = 11.1 Hz), 5.95 (1H, dt-like, *J* = 11.1, 7.7 Hz); ^13^C NMR (75 MHz) δ 12.33 (q), 18.96 (q), 20.37 (t), 22.73 (t), 24.93 (t), 29.04 (t), 30.57 (t), 31.07 (t), 33.66 (t), 36.42 (d), 51.81 (t), 63.23 (d), 71.42 (d), 98.57 (s), 102.01 (s), 109.34 (d), 144.99 (d); MS: 303 (M^+^); HRMS: Calcd for C_19_H_33_NSi 303.2380; Found 303.2372; [α]_D_^26^ –57.9 (*c* 1.11, CHCl_3_).

**(5*R*,8*R*,9*S*)-(-)-5-Hept-4-en-6-ynyl-8-methyloctahydroindolizine (231C)**

To a stirred solution of the acetylene prepared above (20 mg, 0.066 mmol) in MeOH (0.6 mL) was added K_2_CO_3_ (20 mg, 0.15 mmol), and the reaction mixture was stirred at room temperature for 1 h. The reaction mixture was diluted with Et_2_O, and the insoluble material was removed by filtration. The filtrate was evaporated to give indolizidine **231C** (14 mg, 89%) as a colorless oil.

IR (neat) 3312, 2950, 2871, 2778, 1457, 1249, 851 cm^-1^; ^1^H NMR (500 MHz) δ 0.87 (3H, d, *J* = 6.8 Hz), 0.95 (1H, qd, *J* = 12.0, 4.3 Hz), 1.20-1.56 (3H, br m), 1.51-1.57 (7H, br m), 1.60-1.78 (5H, br m), 1.86-1.91 (2H, m), 1.92 (1H, q-like, *J* = 9.0 Hz), 2.28-2.40 (2H, m), 3.08 (1H, br s), 3.26 (1H, t-like, *J* = 8.6 Hz), 5.45 (1H, d-like, *J* = 9.9 Hz), 6.01 (1H, dt-like, *J* = 9.9, 6.7 Hz); ^13^C NMR (75 MHz) δ 18.98 (q), 20.44 (t), 25.02 (t), 29.13 (t), 30.58 (t), 31.26 (t), 33.70 (t), 34.16 (t), 36.63 (d), 51.87 (t), 63.18 (d), 71.29 (d), 80.48 (s), 81.27 (d), 108.15 (d), 145.68 (d); MS: 231 (M^+^); HRMS: Calcd for C_16_H_25_N 231.1986; Found 231.1999; [α]_D_^26^ –107.67 (*c* 0.68, CHCl_3_).

**(5*R*,8*R*,9*S*)-(-)-5-Hepta-4,6-dienyl-8-methyloctahydroindolizine (233D)**

To a stirred solution of **5** (33 mg, 0.1 mmol) in Et_2_O (2 mL) were added NiCl_2_(dppp) (5.4 mg, 0.01 mmol), and then vinylMgBr (1 M in THF, 0.3 mL) at 0 °C, and the resulting mixture was stirred at room temperature for 13 h. The reaction was quenched with satd. NaHCO_3_ (aq) and the aqueous mixture was extracted with Et_2_O (10 mL x 3). The organic extracts were combined, dried over K_2_CO_3_, and evaporated to give a pale yellow oil, which was chromatographed on silica gel (10 g, hexane : acetone = 100 : 1~20 : 1) to afford **233D** (**17**, 30 mg, 86%) as a pale yellow oil.

IR (neat) 3082, 3009, 2927, 2864, 2777, 1457, 996, 901 cm^-1^; ^1^H NMR (500 MHz) δ 0.87 (3H, d, *J* = 6.4 Hz), 0.96 (1H, qd, *J* = 11.5, 3.8 Hz), 1.22-1.42 (4H, br m), 1.44-1.53 (3H, m), 1.61-1.78 (5H, br m), 1.88-1.99 (3H, m), 2.17-2.22 (2H, m), 3.27 (1H, t-like, *J* = 8.1 Hz), 5.08 (1H, d, *J* = 10.2 Hz), 5.19 (1H, d, *J* = 17.1 Hz), 5.44 (1H, q-like, *J* = 8.5 Hz), 6.01 (1H, t, *J* = 10.2 Hz), 6.60-6.68 (1H, m); ^13^C NMR (75 MHz) δ 18.98 (q), 20.42 (t), 25.89 (t), 28.09 (t), 29.09 (t), 31.24 (t), 33.69 (t), 34.17 (t), 36.55 (t), 51.86 (t), 63.38 (d), 71.35 (d), 116.79 (t), 129.24 (d), 132.16 (d), 132.48 (d); MS: 233 (M^+^); HRMS: Calcd for C_16_H_27_N 233.2142; Found 233.2126; [α]_D_^26^ –93.6 (*c* 1.32, CHCl_3_).

**Methyl (5*S*,8*R*,9*S*)-(-)-(8-Ethyl-3-oxooctahydroindolizin-5-yl)acetate (6)**

To a stirred solution of (COCl)_2_ (0.25 mL, 2.82 mmol) in CH_2_Cl_2_ (10 mL) was added DMSO (0.40 mL, 5.63 mmol) at –78 °C, and the resulting mixture was stirred at the same temperature for 5 min. To the reaction mixture was added dropwise a solution of **2** (340 mg, 1.73 mmol) in CH_2_Cl_2_ (3 mL) via a double-tipped stainless steel needle at –78 °C, and the reaction mixture was stirred at –78 °C for 30 min. Triethylamine (1.17 mL, 8.45 mmol) was added to the reaction mixture, and the reaction mixture was warmed to 0 °C for 1 h. The reaction was quenched with H_2_O and the aqueous mixture was extracted with Et_2_O (25 mL x 3). The organic layers were combined, dried, and evaporated to give a pale yellow oil, which was used directly in the next step.

To a stirred solution of the above aldehyde in *t*-BuOH (9 mL) were added 2-methyl-2-butene (7.9 mL, 75.13 mmol), NaH_2_PO_4_•2H_2_O (2.93 g, 18.78 mmol), and then a solution of NaClO_2_ (80%, 1.27 mg, 11.27 mmol) in H_2_O (3 mL) at 0 °C. The reaction mixture was stirred at room temperature for 1 h. and quenched with satd. NaHSO_3_ (aq) at 0 °C. The aqueous mixture was acidified with 10% HCl (aq), saturated with solid NaCl, and extracted with EtOAc (10 mL x 10). The organic extracts were conbined, dried, and evaporated to give a pale yellow oil, which was used directly in the next step.

To a stirred solution of the above carboxylic acid in THF (15 mL) were added Et_3_N (0.29 mL, 2.07 mmol) and ClCO_2_Et (0.2 mL, 2.07 mmol) at 0 °C, and the resulting suspension was stirred at the same temperature for 1 h. The insoluble material was removed by filtration, and the filtrate was evaporated to give a pale yellow oil, which was used directly in the next step.

To a stirred solution of the above oil in Et_2_O (30 mL) was added a solution of CH_2_N_2_ in Et_2_O at 0 °C, and yellow mixture was stirred at room temperature for 16 h. The solvent was removed in vacuo and the residue was dissolved in MeOH (12 mL). To the MeOH solution were added PhCO_2_Ag (43 mg, 0.19 mmol) and Et_3_N (0.52 mL, 3.76 mmol), and the resulting suspension was stirred at room temperature in the dark for 15 h. The insoluble material was removed by filtration, and the filtrate was evaporated to give a pale brown oil, which was chromatographed on silica gel (25 g, hexane : acetone = 30 : 1~10 : 1) to afford **6** (335 mg, 81%) as a pale yellow oil.

IR (neat) 2964, 1739, 1688, 1173 cm^-1^; ^1^H NMR (500 MHz) δ 0.86 (3H, t, *J* = 7.2 Hz), 0.97-1.15 (3H, m), 1.34-1.66 (4H, br m), 1.88-1.94 (1H, m), 2.06-2.21 (1H, m), 2.22-2.27 (2H, m), 2.41 (1H, dd, *J* = 16.5, 4.7 Hz), 3.03 (1H, q-like, *J* = 7.4 Hz), 3.41 (1H, dd, *J* = 16.5, 8.8 Hz), 3.52-3.61 (1H, m), 3.66 (3H, s); ^13^C NMR (75 MHz) δ 10.73 (q), 24.03 (t), 24.19 (t), 28.41 (t), 31.46 (t), 31.84 (t), 37.89 (t), 44.24 (d), 51.39 (q), 52.57 (d), 63.62 (d), 171.97 (s), 175.05 (s); MS: 239 (M^+^); HRMS: Calcd for C_13_H_21_NO_3_ 239.1520; Found 239.1544.

**(5*R*,8*R*,9*S*)-(-)-5-Allyl-8-ethyloctahydroindolizine (7, proposed for 193E)**

To a stirred solution of **6** (167 mg, 0.71 mmol) in THF (8 mL) was added LiAlH_4_ (80 mg, 2.1 mmol) at 0 °C, and the resulting suspension was refluxed for 13 h. After cooling, the reaction was quenched with 10% NaOH (aq), and the aqueous mixture was extracted with CHCl_3_ (10 mL x 10). The organic extracts were combined, dried over K_2_CO_3_, and evaporated to give a colorless oil, which was used directly in the next step.

To a stirred solution of (COCl)_2_ (0.1 mL, 1.14 mmol) in CH_2_Cl_2_ (1 mL) was added DMSO (0.17 mL, 2.40 mmol) at –78 °C, and the reaction mixture was stirred at –78 °C for 5 min. To the resulting mixture was added dropwise a solution of the alcohol obtained above in CH_2_Cl_2_ (2 mL) via a double-tipped stainless steel needle at –78 °C, and the reaction mixture was stirred at –78 °C for 30 min. Triethylamine (0.5 mL, 3.61 mmol) was added to the reaction mixture, and the reaction mixture was warmed to 0 °C for 1 h. The reaction was quenched with satd. NaHCO_3_ (aq), and the aqueous mixture was extracted with CH_2_Cl_2_ (10 mL x 3). The organic layers were combined, dried over K_2_CO_3_, and evaporated to give a pale yellow oil, which was used directly in the next step.

To a stirred suspension of MeP^+^Ph_3_I^-^ (1.27 g, 3.15 mmol) in THF (15 mL) was added a solution of *n*-BuLi (1.6 M in hexane, 1.84 mL, 2.94 mmol) at 0 °C, and the resulting mixture was stirred at the same temperature for 5 min. To the reaction mixture was added dropwise a solution of the aldehyde obtained above in THF (3 mL) via a double-tipped stainless steel needle at 0 °C. The reaction mixture was stirred at room temperature for 17 h., and the reaction was quenched with satd. NaHCO_3_ (aq). The aqueous mixture was extracted with Et_2_O (15 mL x 4), and the organic extracts were combined, dried over K_2_CO_3_, and evaporated to give a pale yellow oil, which was chromatographed on silica gel (20 g, hexane : acetone = 100 : 1~20 : 1) to afford (-)-**7** (proposed for **193E**, 85 mg, 63%) as a pale yellow oil.

IR (neat) 3059, 2963, 2933, 2874, 2779, 910 cm^-1^; ^1^H NMR (500 MHz) δ 0.83-0.92 (1H, m), 0.88 (3H, t, J = 7.7 Hz), 1.03-1.09 (1H, m), 1.13-1.28 (2H, m), 1.41-1.52 (2H, m), 1.57-1.69 (2H, m), 1.74-1.78 (2H, m), 1.85-2.02 (4H, m), 2.08-2.12 (1H, m), 2.44 (1H, m), 3.29 (1H, t, J = 9.0 Hz), 5.00-5.07 (2H, m), 5.77-5.85 (1H, m); ^13^C NMR (75 MHz) δ 11.17 (q), 20.53 (t), 25.99 (t), 29.08 (t), 29.79 (t), 31.21 (t), 39.53 (t), 42.99 (d), 51.95 (t), 63.05 (d), 70.01 (d), 116.37 (t), 135.76 (d); MS: 193 (M^+^); HRMS: Calcd for HRMS: Calcd for C_13_H_23_N 193.1829; Found 193.1833; [α]_D_^26^ –68.7 (*c* 3.30, CHCl_3_).

**(5*R*,8*R*,9*S*)-(-)-8-Ethyl-5-pent-2-enyloctahydroindolizine (221I)**

To a stirred solution of **6** (167 mg, 0.71 mmol) in THF (8 mL) was added LiAlH_4_ (80 mg, 2.1 mmol) at 0 °C, and the resulting suspension was refluxed for 13 h. After cooling, the reaction was quenched with 10% NaOH (aq), and the aqueous mixture was extracted with CHCl_3_ (10 mL x 10). The organic extracts were combined, dried over K_2_CO_3_, and evaporated to give a colorless oil, which was used directly in the next step.

To a stirred solution of (COCl)_2_ (0.1 mL, 1.14 mmol) in CH_2_Cl_2_ (1 mL) was added DMSO (0.17 mL, 2.40 mmol) at –78 °C, and the reaction mixture was stirred at –78 °C for 5 min. To the resulting mixture was added dropwise a solution of the alcohol obtained above in CH_2_Cl_2_ (2 mL) via a double-tipped stainless steel needle at –78 °C, and the reaction mixture was stirred at –78 °C for 30 min. Triethylamine (0.5 mL, 3.61 mmol) was added to the reaction mixture, and the reaction mixture was warmed to 0 °C for 1 h. The reaction was quenched with satd. NaHCO_3_ (aq), and the aqueous mixture was extracted with CH_2_Cl_2_ (10 mL x 3). The organic layers were combined, dried over K_2_CO_3_, and evaporated to give a pale yellow oil, which was used directly in the next step.

To a stirred suspension of *n*-PrP^+^Ph_3_Br^-^ (1.21 g, 3.15 mmol) in THF (15 mL) was added a solution of NaHMDS (1 M in THF, 2.8 mL, 2.8 mmol) at room temperature, and the resulting mixture was stirred at the same temperature for 1 min. To the reaction mixture were added HMPA (0.8 mL), and then dropwise a solution of the aldehyde obtained above in THF (3 mL) via a double-tipped stainless steel needle at -78 °C. The reaction mixture was stirred at –78 °C~room temperature for 22 h., and the reaction was quenched with satd. NaHCO_3_ (aq). The aqueous mixture was extracted with Et_2_O (15 mL x 4), and the organic extracts were combined, dried over K_2_CO_3_, and evaporated to give a pale yellow oil, which was chromatographed on silica gel (20 g, hexane : acetone = 100 : 1~20 : 1) to afford **221I** (96 mg, 62%) as a pale yellow oil.

IR (neat) 3006, 2963, 2932, 2873, 2778, 1459 cm^-1^; ^1^H NMR (500 MHz) δ 0.86-0.91 (1H, m), 0.89 (3H, t, *J* = 7.2 Hz), 0.96 (3H, t, *J* = 7.6 Hz), 1.03-1.09 (1H, m), 1.11-1.27 (2H, m), 1.42-1.52 (2H, m), 1.58-1.70 (2H, m), 1.74-1.82 (2H, m), 1.84-1.97 (3H, m), 2.00-2.15 (4H, m), 2.40 (1H, br), 3.32 (1H, t, *J* = 8.9 Hz), 5.32-5.47 (2H, m); ^13^C NMR (75 MHz) δ 11.17 (q), 14.30 (q), 20.53 (t), 20.78 (t), 26.00 (t), 29.09 (t), 29.82 (t), 31.26 (t), 32.60 (t), 42.96 (d), 51.98 (t), 63.64 (d), 70.06 (d), 125.70 (d), 133.00 (d); MS: 221 (M^+^); HRMS: Calcd for HRMS: Calcd for C_15_H_27_N 221.2142; Found 221.2165; [α]_D_^26^ –77.8 (*c* 3.75, CHCl_3_).

**Electrophysiological recording**

*Xenopus laevis* oocytes were prepared as described previously. [3] In brief, stage V-VI oocytes were isolated and microinjected with cDNAs encoding nicotinic receptor subunit into the nucleus. The α7 subunit cDNA was injected alone, whereas the mixture of α4 and β2 subunit cDNAs was injected in a ratio of 1:1. Oocytes were cultured at 19 ^o^C for 3-7 days. An oocyte was placed in a tube-like chamber in which Ringer’s solution (82.5 mM NaCl, 2.5 mM KCl, 2.5 mM CaCl_2_, 1 mM MgCl_2_, and 5 mM HEPES, pH 7.4) containing 1 μM atropine was perfused by gravity (15 ml/min). Current responses were recorded under two-electrode voltage-clamp method. To apply a pulse of acetylcholine (ACh) to the oocyte, the perfusion fluid was switched to solution containing ACh, using a three-way Teflon solenoid valve (Parker Hannifin Corp., General Valve Division, Fairfield, NJ) controlled by a PC computer with pClamp7 software (Axon Instruments, Union City, CA). A 3-min wash between ACh applications produced reproducible control currents with no desensitization and no residual inward current. Concentration-inhibition curves for alkaloids were analyzed, using Prism software (GraphPad Software, Inc., San Diego, CA). Data were represented as mean ± S.E.M. Animal experiments were performed in accordance with the guidelines approved by the University of Toyama Animal Research Committee.

**Figure legends**

Fig. 1. Inhibitory effect of (-)-**231C** on ACh-induced currents in *X. laevis* oocytes expressing recombinant nicotinic receptors. Currents were recorded in the voltage-clamp mode at -60 mV. Concentrations of ACh used were 1 μM for the α4β2 receptor and 100 μM for the α7 receptor. For test responses, oocytes were preincubated with (-)-**231C** for 3 min and then exposed to ACh with (-)-**231C**. A, representative traces showing the ACh-elicited currents in the absence and presence of (-)-**231C** (3 μM). Horizontal bars indicate the period of perfusion with ACh for 5 s. Vertical scale bars represent 0.5 μA on the α4β2 receptor, and 0.1 μA on the α7 receptor. B, concentration-response curves for (-)-**231C** on recombinant nicotinic receptors. Current responses to ACh in the presence of (-)-**231C** in each oocyte were normalized to the ACh responses (control responses) recorded in the same oocytes. Values represent the mean ± S.E.M. for five to six separate experiments.

Fig. 2. Inhibitory effect of (-)-**221I** on ACh-induced currents in *X. laevis* oocytes expressing recombinant nicotinic receptors. Currents were recorded in the voltage-clamp mode at -60 mV. Concentrations of ACh used were 1 μM for the α4β2 receptor and 100 μM for the α7 receptor. For test responses, oocytes were preincubated with (-)-**221I** for 3 min and then exposed to ACh with (-)-**221I**. A, representative traces showing the ACh-elicited currents in the absence and presence of (-)-**221I** (3 μM). Horizontal bars indicate the period of perfusion with ACh for 5 s. Vertical scale bars represent 0.5 μA on the α4β2 receptor, and 0.1 μA on the α7 receptor. B, concentration-response curves for (-)-**221I** on recombinant nicotinic receptors. Current responses to ACh in the presence of (-)-**221I** in each oocyte were normalized to the ACh responses (control responses) recorded in the same oocytes. Values represent the mean ± S.E.M. for five separate experiments.

Fig. 3. Inhibitory effect of (-)-epi-**193E** on ACh-induced currents in *X. laevis* oocytes expressing recombinant nicotinic receptors. Currents were recorded in the voltage-clamp mode at -60 mV. Concentrations of ACh used were 1 μM for the α4β2 receptor and 100 μM for the α7 receptor. For test responses, oocytes were preincubated with (-)-epi-**193E** for 3 min and then exposed to ACh with (-)-epi-**193E**. A, representative traces showing the ACh-elicited currents in the absence and presence of (-)-epi-**193E** (3 μM). Horizontal bars indicate the period of perfusion with ACh for 5 s. Vertical scale bars represent 0.5 μA on the α4β2 receptor, and 0.1 μA on the α7 receptor. B, concentration-response curves for (-)-epi-**193E** on recombinant nicotinic receptors. Current responses to ACh in the presence of (-)-epi-**193E** in each oocyte were normalized to the ACh responses (control responses) recorded in the same oocytes. Values represent the mean ± S.E.M. for five separate experiments.
